# An artificial intelligence tool for automated analysis of large-scale unstructured clinical cine cardiac magnetic resonance databases

**DOI:** 10.1093/ehjdh/ztad044

**Published:** 2023-07-13

**Authors:** Jorge Mariscal-Harana, Clint Asher, Vittoria Vergani, Maleeha Rizvi, Louise Keehn, Raymond J Kim, Robert M Judd, Steffen E Petersen, Reza Razavi, Andrew P King, Bram Ruijsink, Esther Puyol-Antón

**Affiliations:** School of Biomedical Engineering & Imaging Sciences Rayne Institute, 4th Floor, Lambeth Wing St. Thomas' Hospital Westminster Bridge Road London SE1 7EH; School of Biomedical Engineering & Imaging Sciences Rayne Institute, 4th Floor, Lambeth Wing St. Thomas' Hospital Westminster Bridge Road London SE1 7EH; Department of Adult and Paediatric Cardiology, Guy’s and St Thomas’ NHS Foundation Trust, Westminster Bridge Road, London SE1 7EH, London, UK; School of Biomedical Engineering & Imaging Sciences Rayne Institute, 4th Floor, Lambeth Wing St. Thomas' Hospital Westminster Bridge Road London SE1 7EH; School of Biomedical Engineering & Imaging Sciences Rayne Institute, 4th Floor, Lambeth Wing St. Thomas' Hospital Westminster Bridge Road London SE1 7EH; Department of Adult and Paediatric Cardiology, Guy’s and St Thomas’ NHS Foundation Trust, Westminster Bridge Road, London SE1 7EH, London, UK; Department of Clinical Pharmacology, King’s College London British Heart Foundation Centre, St Thomas’ Hospital, London, Westminster Bridge Road, London SE1 7EH, UK; Division of Cardiology, Department of Medicine, Duke University, 40 Duke Medicine Circle, Durham, NC 27710, USA; Division of Cardiology, Department of Medicine, Duke University, 40 Duke Medicine Circle, Durham, NC 27710, USA; William Harvey Research Institute, NIHR Barts Biomedical Research Centre, Queen Mary University of London, Charterhouse Square, London EC1M 6BQ, UK; Barts Heart Centre, St Bartholomew’s Hospital, Barts Health NHS Trust, W Smithfield, London EC1A 7BE, UK; Health Data Research UK, Gibbs Building, 215 Euston Rd., London NW1 2BE, UK; Alan Turing Institute, 96 Euston Rd., London NW1 2DB, UK; School of Biomedical Engineering & Imaging Sciences Rayne Institute, 4th Floor, Lambeth Wing St. Thomas' Hospital Westminster Bridge Road London SE1 7EH; Department of Adult and Paediatric Cardiology, Guy’s and St Thomas’ NHS Foundation Trust, Westminster Bridge Road, London SE1 7EH, London, UK; School of Biomedical Engineering & Imaging Sciences Rayne Institute, 4th Floor, Lambeth Wing St. Thomas' Hospital Westminster Bridge Road London SE1 7EH; School of Biomedical Engineering & Imaging Sciences Rayne Institute, 4th Floor, Lambeth Wing St. Thomas' Hospital Westminster Bridge Road London SE1 7EH; Department of Adult and Paediatric Cardiology, Guy’s and St Thomas’ NHS Foundation Trust, Westminster Bridge Road, London SE1 7EH, London, UK; Department of Cardiology, Division Heart and Lungs, University Medical Center Utrecht, Utrecht University, 3584 CX Utrecht, the Netherlands; School of Biomedical Engineering & Imaging Sciences Rayne Institute, 4th Floor, Lambeth Wing St. Thomas' Hospital Westminster Bridge Road London SE1 7EH

**Keywords:** Cardiac function, Cardiac magnetic resonance, Quality control, Cardiac segmentation, Artificial intelligence

## Abstract

**Aims:**

Artificial intelligence (AI) techniques have been proposed for automating analysis of short-axis (SAX) cine cardiac magnetic resonance (CMR), but no CMR analysis tool exists to automatically analyse large (unstructured) clinical CMR datasets. We develop and validate a robust AI tool for start-to-end automatic quantification of cardiac function from SAX cine CMR in large clinical databases.

**Methods and results:**

Our pipeline for processing and analysing CMR databases includes automated steps to identify the correct data, robust image pre-processing, an AI algorithm for biventricular segmentation of SAX CMR and estimation of functional biomarkers, and automated post-analysis quality control to detect and correct errors. The segmentation algorithm was trained on 2793 CMR scans from two NHS hospitals and validated on additional cases from this dataset (*n* = 414) and five external datasets (*n* = 6888), including scans of patients with a range of diseases acquired at 12 different centres using CMR scanners from all major vendors. Median absolute errors in cardiac biomarkers were within the range of inter-observer variability: <8.4 mL (left ventricle volume), <9.2 mL (right ventricle volume), <13.3 g (left ventricular mass), and <5.9% (ejection fraction) across all datasets. Stratification of cases according to phenotypes of cardiac disease and scanner vendors showed good performance across all groups.

**Conclusion:**

We show that our proposed tool, which combines image pre-processing steps, a domain-generalizable AI algorithm trained on a large-scale multi-domain CMR dataset and quality control steps, allows robust analysis of (clinical or research) databases from multiple centres, vendors, and cardiac diseases. This enables translation of our tool for use in fully automated processing of large multi-centre databases.

## Graphical Abstract

**Graphical Abstract:**
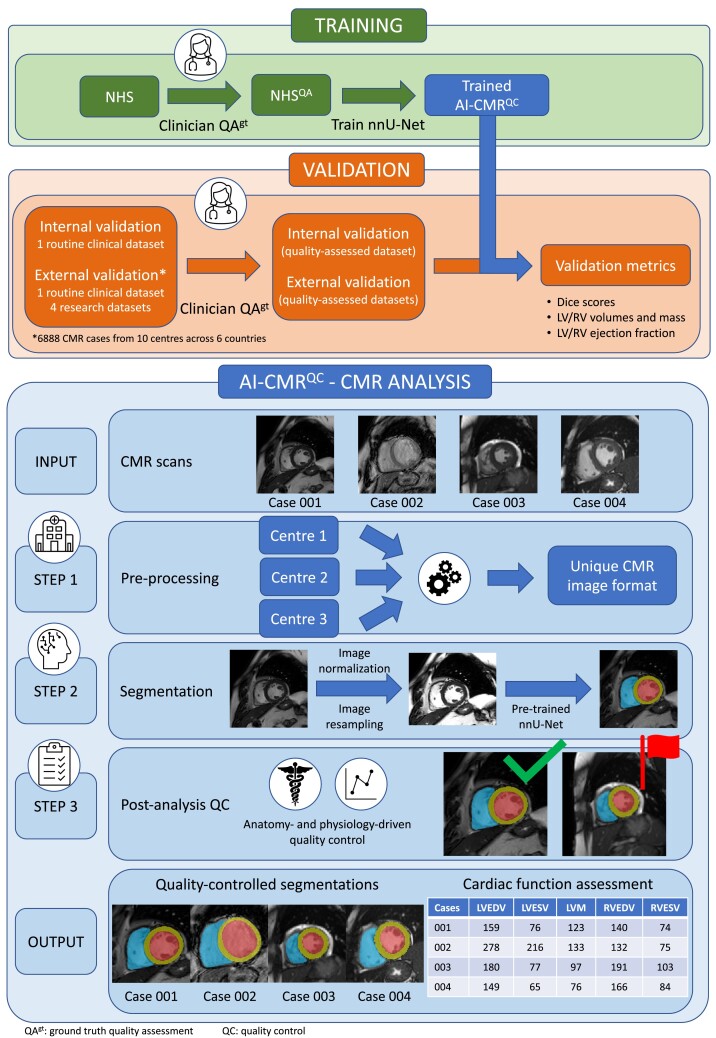
AI-based, quality-controlled CMR analysis tool. The proposed CMR analysis tool consists of CMR image pre-processing steps, an AI method that automatically selects the cine acquisitions prior to image analysis, an AI method that segments the ventricles and the myocardium from short-axis cine CMR stacks, and a post-analysis QC step. QAgt, ground truth segmentation data quality assessment; QC, quality control.

## Introduction

Cardiac magnetic resonance (CMR) is the gold standard for biventricular volume and function quantification as well as myocardial tissue characterization.^1^ In recent years, artificial intelligence (AI) methods have achieved human-level accuracy in the segmentation of short-axis (SAX) cine CMR.^2–6^ A major advantage of AI is the automation of CMR analysis, which could unlock large quantities of new data for clinical research and to inform care. Automated AI-based tools now exist for analysis of clinical CMR at the point of acquisition^4,7^ as well as retrospectively from highly structured and controlled databases (e.g. our previously developed AI-CMR^QC^ tool^2^). However, many large clinical and research CMR databases remain underexploited because of the work involved in making the data suitable for use by AI. A number of tasks are crucial to being able to exploit such data but are often overlooked, for example, selecting the required scans from a wider range of acquisitions, processing the data to ensure consistency of data format/structure, checking the data for quality, and dealing with inconsistencies in image acquisition and labelling protocol (e.g. presence or absence of myocardial segmentations for end-systolic images, including or excluding papillary muscles). In this paper, we address these challenges and, in the process, make two major contributions to the field of AI-based CMR analysis.

## Contributions

Our first major contribution is to present the first example of a fully automated AI-based start-to-end pipeline with quality control (QC) for analysis of large-scale unstructured databases of SAX CMR. To achieve this, we adapt a state-of-the-art AI segmentation model to enable it to deal with the variations in labelling protocol found in many clinical imaging databases. We embed this model into a robust pipeline featuring automated selection of SAX CMR scans, segmentation, and estimation of a range of functional biomarkers followed by automated QC. We train and evaluate the framework on a large and heterogeneous database of CMR scans with ground truth segmentations. This last point is made possible by our second major contribution. We describe the first example of fully automated quality assurance (QA) and correction of *ground truth* segmentations. It is often overlooked that many clinical and research databases feature ground truth segmentations that contain errors. To address this, our QA tools automatically flag cases with potential errors in their ground truth segmentations and even correct some of them automatically.

We perform extensive internal and external validation of our pipeline on datasets featuring a wide range of diseases and scanner types unseen during training, demonstrating that it can automatically process large unstructured databases in a robust way.

## Methods

### Datasets

Our proposed CMR analysis tool was developed and validated using routine clinical CMR scans analysed by expert CMR cardiologists from two NHS hospitals (*n* =3207), which we refer to as the ‘NHS’ dataset. Every CMR study was annotated manually by clinical fellows (*n* = 12), following the standard operating procedures as defined in the SCMR guidelines.^8^ All segmentations were reviewed by level 3 accredited CMR consultants (*n* = 4). The acquisition, pre-processing, and characteristics of this dataset are detailed in Supplementary material online, *Method A* and *Figure S1*. Additionally, the tool was validated externally on the ‘Duke’ clinical dataset from Duke Cardiovascular Magnetic Resonance Center, Durham, NC (*n* = 1319), and on four public datasets of research CMR scans: UK Biobank or ‘UKBB’^9^ (*n* = 4872), ‘ACDC’^10^ (*n* = 150), ‘M&Ms’^11^ (*n* = 375), and ‘M&Ms-2’^12^ (*n* = 360). For all datasets, SAX CMR images acquired at end-diastole (ED) and end-systole (ES) were available. Across the datasets, variation existed in segmentation protocol. All public datasets feature manual or semi-automated segmentations at ED and ES of the right ventricle (RV) blood pool, left ventricle (LV) blood pool, and LV myocardium. However, in both clinical datasets (NHS and Duke), the RV and LV were not always segmented in the same frame and myocardial segmentations were not always present (particularly in ES). In the Duke dataset, papillary muscles were excluded from the LV and RV blood pools whereas in the other datasets, they were included. We note that these types of variation are common in real-world datasets but AI techniques to handle them are currently lacking. The dataset characteristics are summarized in *Table 1*. Additionally, disease and scanner information was stored for each CMR case—where available—to perform a stratified analysis (see Supplementary material online, *Table S1*, for additional information on the different scanners and image acquisition protocols).

**Table 1:** Internal and external validation dataset characteristics

Dataset	Country	Centre	Scanner vendor	Scanner model	Disease
NHS (*n* = 414)	UK	Guy’s and St Thomas’ NHS Foundation Trust	Philips	Achieva 1.5T/3.0T	CHD (*n* = 13) DCM (*n* = 29) IHD (*n* = 15) NOR (*n* = 39) Other (*n* = 50) N/A (*n* = 268)
				Ingenia 1.5T	
			Siemens	Aera 1.5T Biograph mMR	
Duke (*n* = 1319)	US	Duke University Hospital	Siemens	Avanto 1.5T	N/A (*n* = 1319)
				Sola 1.5T	
				Verio 3.0T	
				Vida 3.0T	
UKBB (*n* = 4872)	UK	4 centres	Siemens	Aera 1.5T	NOR (*n* = 4872)
ACDC (*n* = 150)	France	Centre Hospitalier Universitaire Dijon Bourgogne	Siemens	Aera 1.5T	ARV (*n* = 30) DCM (*n* = 30) HCM (*n* = 30) IHD (*n* = 30) NOR (*n* = 30)
				Trio 3.0T	
M&Ms (*n* = 375)	Spain	Clínica Creu Blanca	Canon	Orian 1.5T	AHS (*n* = 3) ARV (*n* = 16) DCM (*n* = 51) HCM (*n* = 103) HHD (*n* = 25) IHD (*n* = 8) LVNC (*n* = 4) NOR (*n* = 125) Other (*n* = 40)
		Hospital Universitari Dexeus	GE	Excite 1.5T	
		Clínica Sagrada Familia	Philips	Achieva 1.5T	
		Hospital Vall d’Hebron	Siemens	Avanto 1.5T	
	Germany	Universitätsklinikum Hamburg-Eppendorf	Philips	Achieva 1.5T	
	Canada	McGill University Health Centre	Siemens	Skyra 3.0T	
M&Ms-2 (*n* = 360)	Spain	Hospital Universitari Dexeus	GE	Excite 1.5T	ARR (*n* = 35) DLV (*n* = 60)^a^ DRV (*n* = 30) HCM (*n* = 60)^a^ CIA (*n* = 35) NOR (*n* = 75)^a^ FALL (*n* = 35) TRI (*n* = 30)
				Explorer 1.5T	
				HDxt 1.5T/3.0T	
		Clínica Sagrada Familia	Philips	Achieva 1.5T	
		Hospital Vall d’Hebron	Siemens	Avanto 1.5T	
				Symphony 1.5T	
				Trio 3.0T	

Dataset names, countries, centres, scanner vendors and models, and cardiac diseases. For the NHS database, the CHD cases consisted of patients with bicuspid aortic valve (*n* = 6), (repaired) tetralogy of Fallot and equivalent (*n* = 2), atrial septal defects (*n* = 2), aortic and pulmonary regurgitation (*n* = 2), and repaired transposition of great arteries (*n* = 1).

AHS, athletic heart syndrome; ARR, congenital arrhythmogenesis; ARV, abnormal right ventricle; CIA, interatrial communication; CHD, congenital heart disease; DCM, dilated cardiomyopathy; DLV, dilated left ventricle; DRV, dilated right ventricle; FALL, tetralogy of fallot; HCM, hypertrophic cardiomyopathy; HHD, hypertensive heart disease; IHD, ischaemic heart disease; LVNC, left ventricular non-compaction; NOR, normal cardiac anatomy and function; TRI, tricuspid regurgitation; N/A, disease information not available for these patients; UKBB, UK Biobank.

These subjects were also included in M&Ms.

### Quality assessment

To ensure the quality of the ground truth data used for training and validation, a thorough data quality assessment (QA^gt^) was applied to all manual segmentations. This process was automated and contained a number of common steps with our QC of model outputs that will be described later. Firstly, to allow validation of the data against clinical metrics, including stroke volume (SV) and ejection fraction (EF), exams in which only one frame (ED or ES) was segmented were excluded. Secondly, we developed an automated algorithm based on data- and knowledge-based criteria to flag anatomical abnormalities in the segmentations (see Supplementary material online, *Method B*). In essence, these criteria check whether the acquisition and/or segmentation covers the heart from the base to the apex, the size of the segmentations, the existence of parts of the segmentation which are disconnected from the RV and LV blood pools, gaps in segmentations, and discordance between LV and RV segmentation size and SV. Thirdly, duplicate segmentations were flagged. Such duplication may occur when the clinician mistakenly uploads the same ground truth segmentation multiple times, or when an original segmentation is corrected by a reviewer.

Subsequent to the automated QA^gt^, all flagged segmentations underwent a manual review by four expert CMR cardiologists to decide inclusion or exclusion of the data from the training and validation data. The outcome of this manual quality assessment of ground truth segmentations (QA^gt^) is detailed in Supplementary material online, *Method C*, and a breakdown of cases excluded by QA^gt^ is reported in Supplementary material online, *Table S2*. Note that this manual review of flagged cases was only necessary to ensure the high fidelity of our training data and is not required when processing test data. The pipeline for processing new test data is fully automated.

### Automated CMR analysis tool

Our proposed CMR analysis tool consists of CMR image pre-processing steps, an AI method that automatically selects the cine acquisitions prior to image analysis, an AI method that segments the ventricles and the myocardium from SAX cine CMR stacks, and a post-analysis QC step (see Graphical abstract). The tool outputs the segmentations for all frames of the full CMR SAX stack together with cardiac biomarkers such as ventricular volumes and EF.

#### Step 1: CMR pre-processing

The first step automatically converts CMR images from their native format to a common format for subsequent analysis (see Supplementary material online, *Method D* and *Table S3*). This is an important but often-overlooked step, as a large variation in file format exists (e.g. different DICOM headers), which challenges the accurate translation of critical metadata, including voxel sizes, slice spacings, and temporal information.

#### Step 2: Data identification

Subsequently, we use our previously published image classification framework^13^ to automatically identify the different cine SAX CMR sequences from the data and format them into a unified structure for analysis. This step was only performed for the two clinical databases, NHS and Duke, as the other databases only provided the SAX CMR sequence.

#### Step 3: CMR segmentation

The LV and RV endocardia and the LV myocardium are segmented from SAX cine CMR using ‘nnU-Net’, a state-of-the-art medical imaging segmentation framework.^14^ nnU-Net aims at reducing the effect of heterogeneities inherent in imaging data (in this study, CMR data) from different clinical centres, MRI vendors, or imaging protocols. To do so, nnU-Net automatically adapts its image pre-processing (*z*-score intensity normalization and image resampling), network architecture, and hyperparameters to any given image dataset. These strategies allow nnU-Net to outperform most AI methods (even highly specialized ones) in international medical image segmentation challenges.^14^

Additionally, we modified nnU-Net’s loss function to tackle the problem of inconsistent image labelling protocols. For example, in most clinical contexts, the myocardium is only segmented at the ED but not in the ES frame. More details on nnU-Net and our loss function adaptation are provided in Supplementary material online, *Method E*.

A further step was implemented to allow exclusion of papillary muscles from the LV and RV blood pool segmentations using Otsu’s threshold method^15^ (see Supplementary material online, *Method F*, for more details). This step can be applied per clinician preference. Here, it was used for the Duke dataset, to cater for the segmentation protocol followed when forming the ground truth data for the Duke dataset (see Supplementary material online, *Figure S2*).

#### Step 4: Post-analysis QC

AI algorithms typically perform well in pixelwise segmentation of images but lack biophysical/anatomical constraints. This means that segmentation outputs are not always realistic. For example, a cardiac segmentation algorithm could output a result that includes an additional region of blood pool/myocardium outside of the heart (e.g. in the stomach area, which can appear like a muscular wall with fluid inside) or create a hole in the LV myocardial lateral wall. We developed a set of automated QC steps (note the distinction between QA, which is an automated series of checks applied to the *training* data of the framework, and QC, which is automated checking of model *outputs*.) that detect and potentially address these errors in segmentations, using prior knowledge of the anatomy and physiology of the heart. A number of these steps also feature in the QA^gt^ of the databases used for model training (see Supplementary material online, *Methods B* and *G*). On the output of the CMR segmentation model, the post-analysis segmentation QC detects segmentations that (i) are discontinuous (e.g. non-segmented slices between segmented slices), (ii) do not adhere to the anatomical relationship between the ventricles and the myocardium, and (iii) are disconnected from the rest of the heart. See Supplementary material online, *Method G*, for details. Where possible, segmentations were automatically adapted after being flagged by these QC steps (e.g. segmentations outside the heart area were automatically deleted). More complex issues (for example, gross distortions in anatomy or large differences in (stroke) volumes between ventricles) were not addressed. However, those cases were still flagged by the QC step for clinician review (see Supplementary material online, *Method G* and *Table S4*).

### Training

We trained a 2D nnU-Net on a subset of randomly selected cases from the NHS dataset (*n* = 2793) using five-fold cross validation on the training set. Subsequently, nnU-Net was applied as an ensemble of the five models resulting from this cross validation.

### Validation and statistical analysis

We performed an internal validation of our tool using the remaining NHS cases that were not used for training (*n* = 414). Subsequently, we performed an external validation in five additional datasets (*n* = 6888) which included clinical CMR scans of patients with a range of diseases acquired at 10 international centres using 1.5 and 3T CMR scanners from all major vendors (Canon Medical Systems Corporation, Otawara, Tochigi, Japan; General Electric Healthcare, Chicago, Illinois, USA; Philips Healthcare, Best, the Netherlands; Siemens Healthineers, Erlangen, Germany). In this manuscript, external databases are considered to be those from different centres from the data used for training the model.

Dice scores were used to assess the agreement between the automated and manual segmentations. A Dice score of 0% indicates no agreement, and a Dice score of 100% indicates perfect agreement. Distributions of Dice scores were tested for symmetry using D’Agostino’s *K*^2^ test and are reported using median (interquartile range) values. To provide more clinically meaningful validation, we used Bland–Altman plots to compare the LV and RV end-diastolic volume (EDV), end-systolic volume (ESV), EF, and left ventricular mass (LVM) obtained using our method vs. manual analysis and calculated absolute errors (i.e. |automated−groundtruth|) for each metric. See Supplementary material online, *Method H*, for more details of the computation of the cardiac biomarkers.

Mann–Whitney *U* tests were performed to compare both the Dice scores and the cardiac biomarkers between the NHS validation cases and the external validation datasets. Finally, we used box plots to compare performance over major groups of cardiac diseases and scanner vendors for CMR cases. Wilcoxon signed-ranked tests were applied to determine whether there were significant errors in biomarker estimation for the different disease and CMR vendor groups. *Post hoc* Bonferroni correction for multiple comparisons was performed for all statistical tests.

We additionally compared the segmentation performance of our tool against human inter-observer variability. To do so, a set of 50 subjects from the NHS validation dataset was randomly selected and each subject was analysed by three level 3 accredited CMR clinicians (O1, O2, and O3) independently. The difference of clinical measurements was evaluated between each pair of observers (O1 vs. O2, O2 vs. O3, and O3 vs. O1) and between manual and automated analyses (see Supplementary material online, *Table S5*).

### Comparison to other state-of-the-art methods

We compare the proposed method with the original nnU-Net AI trained model on the M&Ms dataset^11,16^ and tested on the internal NHS validation dataset. For validation, we computed the Dice scores between the automated and manual segmentations.

## Results

### Data identification

For the two clinical databases (internal NHS dataset and the Duke database), our image classification framework^13^ correctly detected the cine SAX sequence in 100% of cases.

### Dice

For the internal NHS dataset, median Dice scores between the automated and manual segmentations were 94.3% for the LV blood pool (LVBP), 85.5% for the LV myocardium (MYO), and 90.8% for the RV blood pool (RVBP). For the external datasets, median Dice scores of the automated compared with manual segmentations were >91.3% for the LVBP, >83.0% for the MYO, and >87.4% for the RVBP. Dice scores for each dataset and cardiac label are shown in *Table 2*. Additionally, Dice scores for each scanner model and cardiac label are shown in Supplementary material online, *Table S6*.

**Table 2:** Dice scores of automated vs. manual segmentations

Dataset	LVBP (%)	MYO (%)	RVBP (%)	Average (%)
NHS	94.3 (4.0)	85.5 (4.4)	90.8 (5.4)	91.3 (7.5)
Duke	91.3 (6.5)***	83.0 (4.8)***	89.3 (6.8)***	89.2 (8.3)***
UKBB	91.8 (5.9)***	83.0 (6.5)***	87.8 (7.8)***	87.4 (8.9)***
ACDC	95.5 (3.9)	87.4 (3.5)***	91.8 (7.0)*	90.6 (7.9)
M&Ms	93.4 (5.4)***	85.4 (5.7)	90.4 (6.8)	89.4 (8.3)***
M&Ms-2	94.6 (4.2)	86.0 (5.6)*	90.9 (6.7)	90.3 (8.3)**

Dice scores are shown as median (interquartile range) percentages for each validation dataset, including values per label and their average. Comparisons between the Dice scores of the NHS validation cases and the external validation datasets were performed using Mann–Whitney *U* tests. Pairwise *post hoc* testing was performed using Bonferroni correction for multiple comparisons. Asterisks indicate statistically significant differences for each label after correction (20 tests), where *P < 0.01/20, **P < 0.001/20, and ***P < 0.0001/20.

LVBP, LV blood pool; MYO, LV myocardium; RVBP, RV blood pool.

### Cardiac biomarkers

**Figure 1:**
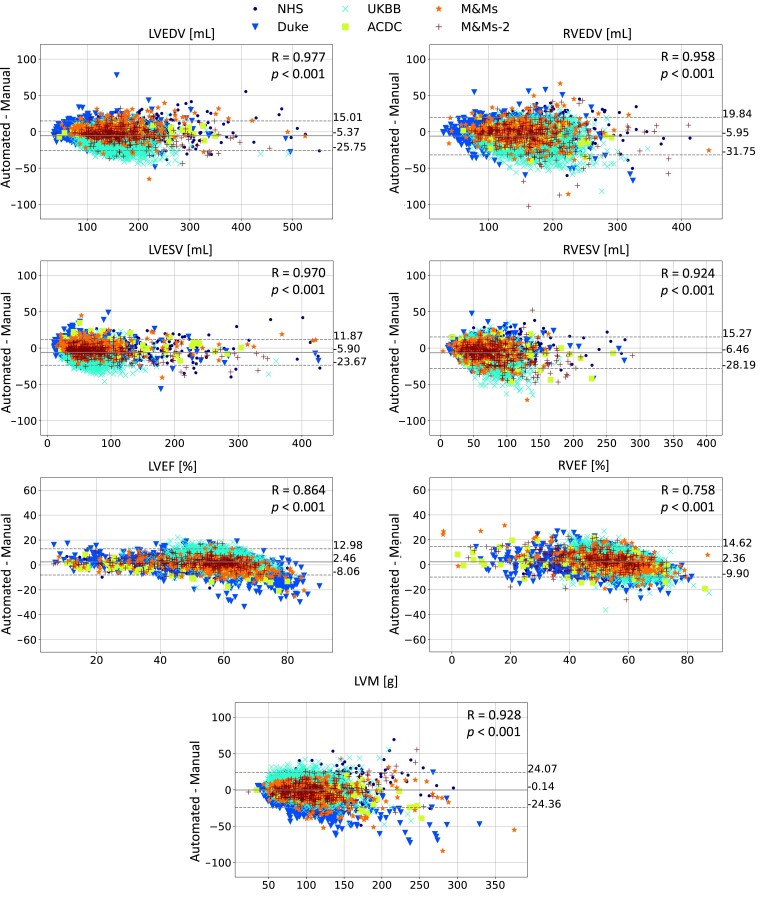
Bland–Altman analysis of cardiac volume, ejection fraction, and mass: Cardiac biomarkers derived from manual and automated segmentations were compared for all validation cases. The thick line depicts the mean bias between the automated and manual analyses. The top and bottom dotted lines correspond to +1.96 and −1.96 standard deviations from the mean bias, respectively. The Pearson’s correlation coefficients (*R*) between our method and the manual analysis (and the corresponding *P*-values) are indicated for each cardiac biomarker. LVEDV, left ventricular end-diastolic volume; LVESV, left ventricular end-systolic volume; LVEF, left ventricular ejection fraction; LVM, left ventricular mass; RVEDV, right ventricular end-diastolic volume; RVEF, right ventricular ejection fraction; RVESV, right ventricular end-systolic volume.

A Bland–Altman analysis of the differences between cardiac biomarkers derived from the manual and automated segmentations is shown in *Figure 1*. For the internal NHS dataset, median absolute errors in cardiac biomarkers were 6.7 mL for LVEDV, 6.3 mL for LVESV, 3.4% for LVEF, 8.9 g for LVM, 8.5 mL for RVEDV, 6.4 mL for RVESV, and 4.2% for RVEF.

For the external datasets, median absolute errors in cardiac biomarkers were <7.3 mL for LVEDV, <8.4 mL for LVESV, <5.3% for LVEF, <13.3 g for LVM, <9.2 mL for RVEDV, <8.9 mL for RVESV, and <5.9% for RVEF. There was no significant bias for cardiac volumes or EF for the internal or external databases.

**Figure 2:**
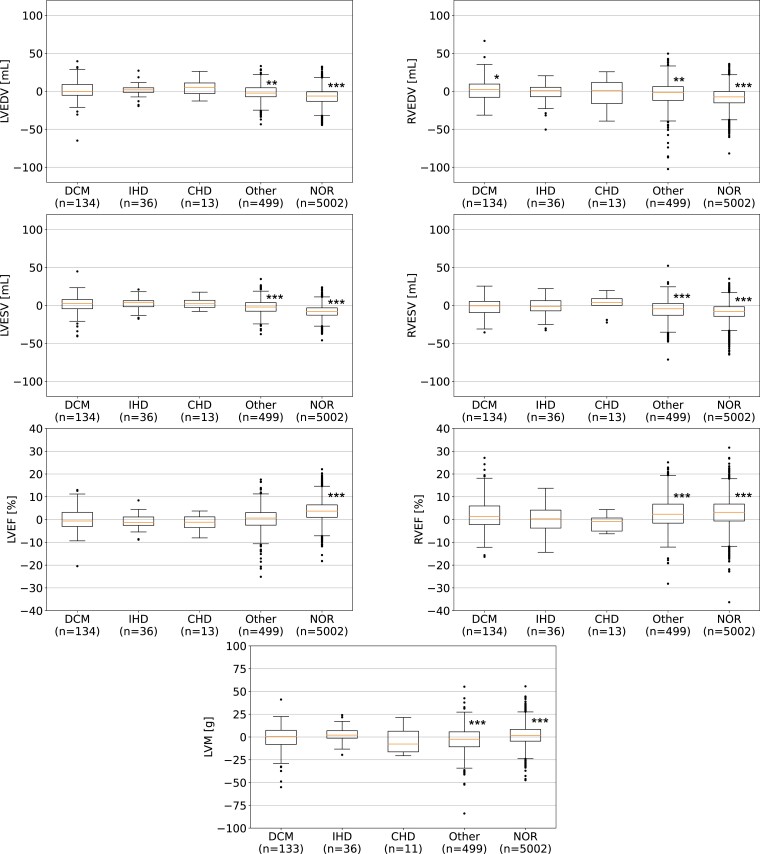
Box plots of manually and automatically derived cardiac biomarkers for each disease group: Statistical differences from zero were assessed using Wilcoxon signed-rank tests. Pairwise *post hoc* testing was performed using Bonferroni correction for multiple comparisons. Asterisks indicate statistically significant differences from zero for each group after correction (five tests), where *P < 0.01/5, **P < 0.001/5, and ***P < 0.0001/5. CHD, congenital heart disease; DCM, dilated cardiomyopathy; IHD, ischaemic heart disease; LVEDV, left ventricular end-diastolic volume; LVESV, left ventricular end-systolic volume; LVEF, left ventricular ejection fraction; LVM, left ventricular mass; NOR, normal cardiac anatomy and function; Other, other cardiac diseases; RVEDV, right ventricular end-diastolic volume; RVEF, right ventricular ejection fraction; RVESV, right ventricular end-systolic volume.

**Figure 3:**
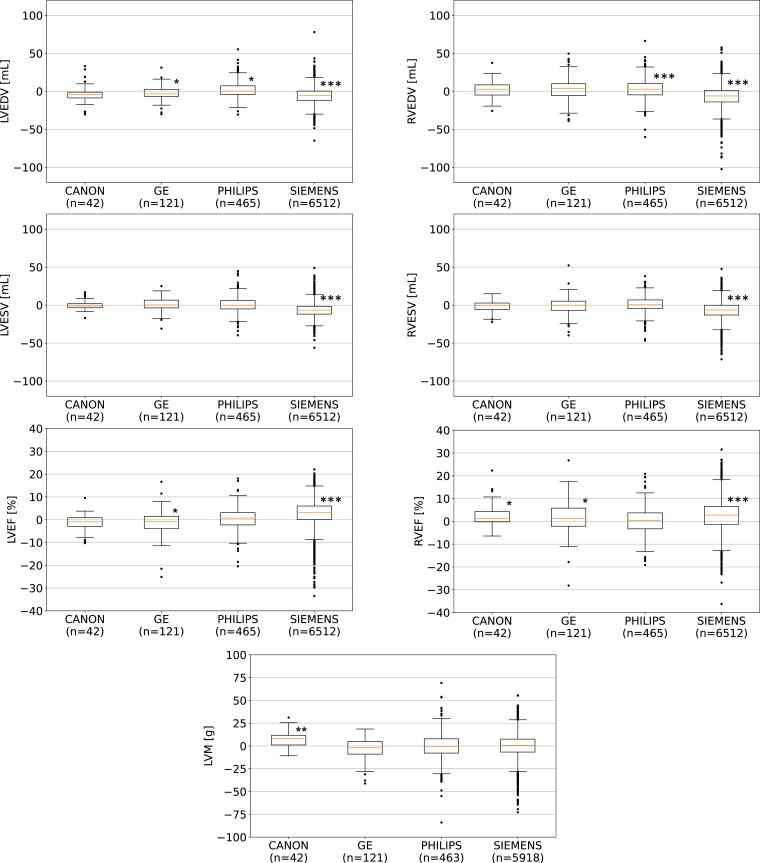
Box plots of manually and automatically derived cardiac biomarkers for each scanner group: Statistical differences from zero were assessed using Wilcoxon signed-rank tests. Pairwise *post hoc* testing was performed using Bonferroni correction for multiple comparisons. Asterisks indicate statistically significant differences from zero for each group after correction (four tests), where *P < 0.01/4, **P < 0.001/4, and ***P < 0.0001/4. CHD, congenital heart disease; DCM, dilated cardiomyopathy; IHD, ischaemic heart disease; LVEDV, left ventricular end-diastolic volume; LVESV, left ventricular end-systolic volume; LVEF, left ventricular ejection fraction; LVM, left ventricular mass; NOR, normal cardiac anatomy and function; Other, other cardiac diseases; RVEDV, right ventricular end-diastolic volume; RVEF, right ventricular ejection fraction; RVESV, right ventricular end-systolic volume.

*Table 3* shows, for each dataset, the ground truth values (first row) and the median (interquartile range) absolute errors (second row) of the automated analysis. There are statistically significant differences in performance between the internal NHS validation cases and the external validation datasets. The box plots in *Figures 2* and *3* show the errors between ground truth and automated cardiac biomarkers grouped by cardiac disease and scanner type, with the largest errors found in healthy (NOR) subjects and Siemens scanners, respectively. Similar box plots grouped by magnetic field strength are shown in Supplementary material online, *Figure S3*.

**Table 3:** Errors between cardiac biomarkers derived from automated and manual segmentations

	LV				RV		
Dataset	EDV (mL)	ESV (mL)	EF (%)	LVM (g)	EDV (mL)	ESV (mL)	EF (%)
NHS	198.8 (75.3)	108.9 (70.3)	48.7 (14.0)	116.5 (42.6)	172.4 (52.4)	84.6 (40.3)	52.3 (9.8)
	6.7 (13.5)	6.3 (12.9)	3.4 (4.3)	8.9 (17.9)	8.5 (15.8)	6.4 (12.5)	4.2 (5.4)
Duke^a^	107.7 (46.6)	50.7 (38.2)	55.9 (14.8)	107.4 (46.1)	104.6 (41.4)	52.6 (28.3)	51.0 (11.2)
	5.7 (11.0)***	5.0 (9.7)	5.3 (7.1)***	13.3 (17.2)***	6.0 (11.8)***	4.5 (9.1)***	5.2 (6.8)**
UKBB	149.0 (34.9)	62.0 (20.7)	58.8 (6.3)	91.9 (25.4)	155.6 (37.5)	69.1 (22.7)	56.1 (6.4)
	7.3 (12.5)***	8.4 (9.6)***	4.7 (4.2)***	6.6 (12.8)**	9.2 (14.7)***	8.9 (12.5)***	5.3 (5.9)***
ACDC	161.4 (67.6)	94.0 (71.0)	47.6 (18.8)	126.7 (50.1)	151.7 (51.2)	86.1 (51.1)	46.1 (18.1)
	3.6 (5.8)	5.3 (8.0)***	4.6 (5.1)***	6.8 (12.7)***	6.1 (11.2)***	5.7 (11.1)**	5.6 (7.6)
M&Ms	159.1 (61.1)	73.0 (54.2)	56.9 (13.7)	115.9 (49.1)	147.4 (49.9)	69.5 (37.4)	53.9 (12.2)
	6.2 (13.4)	5.1 (9.8)***	4.2 (5.4)***	8.2 (16.6)***	8.4 (16.9)	5.9 (13.7)***	5.6 (7.3)***
M&Ms-2	174.9 (61.6)	89.6 (58.9)	52.0 (13.7)	112.0 (36.7)	169.1 (56.5)	89.5 (42.1)	48.3 (12.8)
	6.3 (9.6)***	5.5 (8.4)***	3.4 (4.2)	7.3 (14.2)***	8.7 (16.5)***	8.3 (13.9)***	5.9 (7.0)***

For each validation dataset, the first row (highlighted in grey) reports the ground truth clinical measurements for each cardiac biomarker as median (interquartile range). The second row reports the median absolute errors (interquartile range) between cardiac biomarkers derived from the automated and ground truth segmentations. Comparisons between the errors of the NHS validation cases and the external validation datasets were performed using Mann–Whitney *U* tests. Pairwise *post hoc* testing was performed using Bonferroni correction for multiple comparisons. Asterisks indicate statistically significant differences for each biomarker after correction (35 tests), where *P < 0.01/35, **P < 0.001/35, and ***P < 0.0001/35.

LV, left ventricle; RV, right ventricle; EDV, end-diastolic volume; ESV, end-systolic volume; EF, ejection fraction; LVM, left ventricular mass.

Lower ventricular volumes in the Duke dataset are due to the exclusion of papillary muscle from the ground truth segmentations.

To validate the impact of our automated checking of ground truth segmentations (QA^gt^), Supplementary material online, *Table S7*, shows a comparison for the internal NHS validation dataset between the proposed method with and without QA^gt^. The results demonstrate the importance of performing stringent automated data quality assessments on training databases. Supplementary material online, *Table S7*, also shows results for a nnU-Net model trained only using the M&Ms database, demonstrating the necessity to train CMR segmentation models on large and heterogeneous databases.

### Quality control

From the test set segmentations of the CMR segmentation model, 607 cases out of 7302 were flagged, of which 18 were automatically adapted. The remaining cases that were flagged were not adapted, and all were included in the segmentation and biomarker estimation results presented above. For the internal database, 6.59% of cases were flagged, and on average, for the external datasets, 7–15% of cases were flagged (see Supplementary material online, *Table S4*). To validate the impact of our automated QC of model outputs, Supplementary material online, *Table S8* and *Table S9*, show the improvement in Dice scores and errors in ventricular volume quantification with and without post-analysis QC. For all labels (LV blood pool, LV myocardium, and RV blood pool), Dice scores improved with post-analysis QC. The error in ventricular volumes also decreased. The largest decreases were seen for LVM and RVESV.

## Discussion

In this paper, we have proposed and validated the first start-to-end pipeline for fully automated analysis of large, unstructured clinical and research databases of CMR scans. This work addresses two majors but often-overlooked challenges in the exploitation of large CMR databases. First, data are often stored in an inconsistent and/or unstructured manner (e.g. different file format/structure and different segmentation protocols). Second, ground truth data often contain errors. We have proposed AI tools that address these challenges and hence enable for the first time fully automated processing of large databases of SAX CMR scans as well as flagging and potentially correcting error cases. Furthermore, our framework incorporates automated QC of its outputs, enabling the AI to ‘know when it has failed’, which is an important characteristic for full automation of routine but laborious clinical tasks.

We have validated our tool on a total of 7293 CMR scans, including on two large clinical datasets containing routine CMR exams (*n* = 1270 and *n* = 414) and on four external research datasets (*n* = 4787, *n* = 345, *n* = 345, and *n* = 132). Through this extensive validation, we have shown that our tool achieves human-level accuracy for SAX cine CMR segmentation across a wide range of diseases, vendors, and clinical imaging protocols. Our method yielded median Dice scores of >91%, >83%, and >87% for the LVBP, LVM, and RVBP, respectively, translating into median *absolute errors* in cardiac biomarkers of <8.4 mL for the LV, <9.2 mL for the RV <13.3 g for the LVM, and <5.9% for EF across all datasets. Our results (*Table 3*) did show a statistically significant difference in the accuracy of some clinical biomarkers between the internal and external validation databases. However, the limits of agreement between manual assessment and our AI tool for cardiac volumes was similar to the inter-observer variability observed in the analysis of a sample of our own data by three independent clinical experts (see Supplementary material online, *Table S5*) and inter-observer variability values reported in the literature.^7,17–19^ Note that it is to be expected that our method would not surpass these limits of agreement between observers, as our ground truth data was segmented by a range of different experts from different institutions.

**Figure 4:**
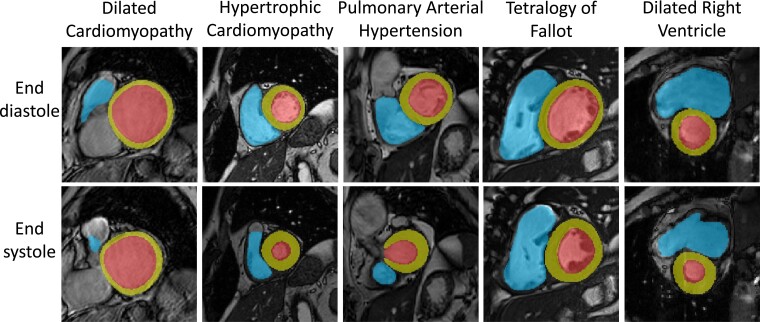
Examples of automated segmentations from different disease groups: The middle slice of each automated segmentation is shown in end-diastole (ED) and end-systole (ES). Cardiac labels: LV blood pool (red), LV myocardium (yellow), and RV blood pool (blue).

We examined the performance of our method over different phenotypes of cardiac disease (see *Figure 2*). Performance was good across different disease groups, including the spectrum of cardiomyopathic diseases, in addition to several congenital cases such as pulmonary arterial hypertension and tetralogy of Fallot (see *Figure 4* for examples). Although the errors were significantly different from zero in some groups, they were within the ranges of inter-observer variability in manual CMR analysis^7,17–19^ and are therefore similar to those experienced in clinical practice; the worst-performing phenotype (healthy subjects—NOR category in *Figure 1*) had median absolute errors of <10 mL for volumes and <5% for EF. Most healthy subjects came from the UK Biobank dataset, whose segmentations were obtained using a stringent standardized operating procedure whereby each analyst was specifically trained to limit inter-observer variability, thus leading to a systematic segmentation style. This could explain why this group had the highest bias in our analysis. The training dataset (NHS) is likely to reflect the more typical level of inter-observer variability among CMR cardiologists, which also exists in all validation datasets except for the UK Biobank. As an adult congenital heart disease (CHD) list is part of the routine CMR service of the NHS hospitals, some (but a limited number of) CHD cases were included in training and testing. This included patients with (repaired) tetralogy of Fallot, transposition of the great arteries, bicuspid aortic valves, and pulmonary valve diseases. Patients with single-ventricle circulation were excluded. An analysis of congenital disease phenotypes is beyond the scope of the current work but will be subject of a future study. Overall, these results show that our tool performs well for a typical (non-congenital) CMR list.

Our method also performed well across all scanner types (see *Figure 3*), including those not seen during training (Canon and GE). The largest errors were found in Siemens data, but this error was again within inter-observer variability in CMR analysis.^18,19^ Again, this is likely explained by the systematic difference in segmentation style between the UK Biobank data—which was acquired using a single Siemens scanner model—and the NHS data.

We deliberately trained the segmentation model on one clinical dataset (the NHS dataset) alone, without using the full variety of scanner vendors and protocols in the external datasets. This reflects the real-world challenge of application of AI tools in previously unseen data, due to protocol and scanner updates. The good domain generalizability of our method to unseen data in the validation experiments demonstrates the strength of our framework in generalizing to data unseen during training.

A number of previous studies have evaluated AI segmentation models on CMR data, although typically on smaller/non-existent and/or less variable external validation sets. Nevertheless, the performance of our method is comparable to previously published algorithms, e.g. see^2,4–6,10,20–22^ and the references therein. Furthermore, we have compared the proposed method with the original nnU-Net AI trained model on the M&Ms dataset (see Supplementary material online, *Table S7*) and show an improved performance.

### Novelty of our method

Over the last years, many research and commercial solutions have been proposed for automated segmentation of LV and RV volumes from SAX cine CMR sequences. However, these methods are only suitable for analysis of data at the point of acquisition (where manual QC can be routinely performed) or of highly structured databases such as the UK Biobank. Such databases are relatively uncommon, but our AI-based pipeline enables fully automated analysis of a wider range of clinical databases, which are typically unstructured and/or contain errors. Furthermore, most existing automated CMR analysis methods have been developed and validated on a single highly controlled dataset, with limited external validation.^2,6^ One recent study used multi-centre, multi-vendor data for training^4^; however, it did not include Canon scanners and only validated externally using a single-centre, single-scanner dataset. AI methods tend to not be domain agnostic and can generalize poorly to other domains.^23^ This is an issue not only in research but also affects algorithms already being deployed in commercial CMR analysis software packages.^17^ Therefore, extensive external validation of new AI algorithms in large, heterogeneous datasets that include a range of diseases is an important step to perform prior to clinical translation. In this study, we have aggregated CMR data from two UK hospitals, one US hospital, and several external research datasets to perform such a validation.

We also stratified our models’ validation experiments over groups of patients with different disease phenotypes. This is another important aspect of validation of clinical AI tools, as errors in segmentation algorithms might impact some disease phenotypes more than others.

Altogether, the new contributions in this paper, combined with our previous developments for automated detection of cine CMR views from full CMR scans^13^ and post-analysis QC of the output parameters for physiological feasibility,^2^ show that our framework is a robust tool for automated analysis of large, unstructured databases of CMR scans. It can automatically identify CMR scans from a file/folder structure (even if this structure includes other data), detect target cine CMR sequences for analysis, analyse these images, and provide parameters of biventricular function accurately and robustly for all major disease groups, scanner vendors, and imaging protocols, while flagging cases with potential errors in data.

Finally, we are in the process of making this tool available as an easy-to-use web application which will be accessible for external researchers.

## Quality-controlled AI

Most commercial cardiac analysis software has already implemented AI algorithms. For example, a recent study^7^ compared the performances of three different commercial solutions [CardioAI (Arterys), CVI42 (Circle Cardiovascular Imaging), and SuiteHeart (Neosoft)] in a cohort of 200 ischaemic heart disease patients. Although they show excellent agreements with manual annotation, most of these algorithms are still not optimized to generalize to out-of-distribution data and suffer from reduced performance when applied to other datasets.^7,23^ Therefore, these tools require continuous clinician oversight, which is sufficient and desirable for prospective clinical reporting. However, in the current era of big data, AI tools that can analyse large (retrospective) datasets or registries robustly are essential for developing clinical research. In the current work, we detect and automatically correct some common errors in segmentations (e.g. additional segmented regions outside of the heart area). More complex distortions were not addressed, but together with our previously developed post-analysis QC steps of the obtained ventricular volumetrics,^2^ the overall QC process can be used to flag potential errors during automated analysis to clinicians for review when the tool is implemented for use. This aids a trustworthy and transparent system.

The beneficial effect of post-analysis QC and automated adaption is demonstrated by the improvements in Dice scores and volume errors when comparing the AI algorithms’ output with and without QC shown in Supplementary material online, *Tables S8* and *S9*. Dice scores and the absolute error in volume quantification between AI and manual assessment improved for all cardiac metrics. The largest improvements were found for LVM and RV parameters. LVM and RV anatomy are known to be the most challenging tasks for AI algorithms, and these improvements are therefore clinically relevant.

The post-analysis QC flagged 6–15% of cases from the output of the segmentation model in the different test datasets (see Supplementary material online, *Table S4*). The highest rates were in the ACDC and M&Ms databases, and nearly all flags related to an SV difference >25%. This was expected, as many of the cardiomyopathy patients in these datasets suffer from valvar regurgitation. Flagged cases therefore do not always reflect errors in the results but aid to highlight challenging/unusual cases during large database processing for clinicians for review.

Other techniques have been proposed in the literature for QC of segmentation model outputs, and these could also be incorporated into our framework if they brought added value. For example, Robinson *et al.*^24^ proposed to use an atlas registration-based approach to estimate segmentation quality. Uncertainty-based approaches to segmentation QC have been proposed by Puyol Anton *et al*.^3^ and Arega *et al*,^8^ both for segmentation of T1 mapping CMR images.

### The importance of ground truth data quality

**Figure 5:**
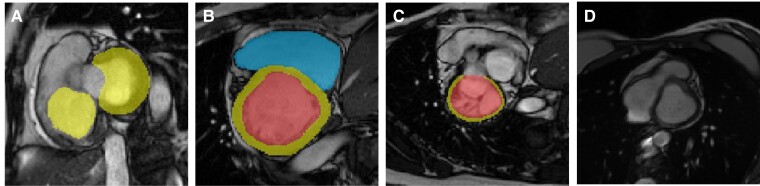
Examples of erroneous ground truth segmentations identified during manual QA^gt^. Cardiac labels: LV blood pool (red), LV myocardium (yellow) and RV blood pool (blue). (*A*) Image in ED: note that the LV myocardium is segmented but the LV blood pool segmentation is absent and that the RV segmentation is labelled as myocardium (yellow); (*B*) top slice of the cine stack in ED: the basal part of the heart is not included in the cine SAX stack; (*C*) image in ED: note the unusual LV structure that was segmented and the absence of an RV segmentation; (*D*) image in ES; note the absence of LV and RV segmentations, while myocardium is present for both.

The quality of the ground truth segmentations is important when developing and evaluating AI methods, but most existing works assume that ground truth quality is high without checking. Therefore, we performed automated data quality assessments (QA^gt^) on all datasets used for training and validation. The flagging of potential erroneous segmentations was fully automated and followed by a manual inspection by expert cardiologists if the error could not be corrected automatically. Through this QA^gt^, we excluded a number of erroneous ground truth segmentations (e.g. a partial segmentation that was stored halfway through the manual analysis and was never completed by the clinician) from the clinical datasets. However, perhaps surprisingly, a number of cases from the external validation datasets (i.e. ACDC, M&Ms, and M&Ms-2) were also excluded, as the segmentation quality of these cases did not adhere to the clinical standards for ventricular segmentation for volume quantification published by the European and American CMR societies.^25,26^ The majority of these errors consisted of the absence of the basal slices in the SAX cine acquisition (see examples of erroneous ground truth segmentations in *Figure 5*). Since the basal slices are challenging for segmentation algorithms, missing basal slices have direct implications on validation results. Another common challenge was the segmentation of the RV base. Segmentations did not always continue until the pulmonary valve as is recommended in the standards of segmentation by the European and American CMR societies^25,26^ (see *Figure 5B* which shows a ground truth segmentation missing the top basal slice).

Our QA^gt^ of the ground truth segmentations from the external validation datasets led to the exclusion of 1.4% of cases (see Supplementary material online, *Table S2*). While this number is relatively small, we argue that assuring high standards for quality of ground truth data is important when training AI algorithms. Supplementary material online, *Figure S4*, shows examples of cases flagged by QA^gt^ for the external validation datasets and Supplementary material online, *Method C*, includes the list of all cases excluded from the online available databases (ACDC, M&Ms, and M&Ms2). Supplementary material online, *Table S7*, shows that QA^gt^ improves the robustness of the resulting trained segmentation model. Note that, for completeness and to allow evaluation against previously published results, we have included the performance of our method on the full original external validation datasets (i.e. without QA) in Supplementary material online, *Tables S8* and S*9*.

In our work, we have proposed to use a set of heuristic rules for QA of segmentations. However, in principle, other QA approaches could be substituted within our framework. For example, the recent literature has proposed a range of approaches for image QA, including methods based on hand-crafted quality metrics^27^ and deep learning–based methods^28^ for detecting predefined types of image artefacts. Methods proposed for QA of segmentations include the supervised approach of Fournel *et al.*^29^ who proposed to directly predict 2D and 3D Dice scores from segmentation/image pairs and the unsupervised approach of Galati and Zuluaga *et al.*^30^ who used the reconstruction error of a convolutional autoencoder trained on ground truth segmentations as a surrogate measure of segmentation quality. All of these approaches could in principle be added to our framework, and in the future, we will investigate if they bring added benefit over our current approach.

### Dealing with papillary muscles

Different protocols are used when segmenting SAX cine CMR images. Some CMR departments include papillary muscles in the LV and RV blood pools, while others exclude them. This depends both on clinician preference and on the segmentation technique used (e.g. drawing myocardial borders manually or semi-automatically using region growing).^26^ Our tool was originally trained to include papillary muscles in the ventricular blood pools. However, papillary muscles were excluded from the segmentations in the Duke external validation dataset. Therefore, we developed and applied an automatic image thresholding technique—based on Otsu’s method—to apply to the segmented blood pools to obtain segmentations that exclude the papillary muscles. This not only allowed external validation on the Duke dataset but also allows users of our tool to choose their preferred segmentation strategy.

### Limitations

Although we trained our segmentation algorithm on a large clinical dataset (*n* = 2793) and showed good generalization to out-of-distribution data, the validation data did not include all variations of data in clinical practice. In particular, complex congenital heart diseases were absent from our training data. Similarly, our five external validation datasets are not all encompassing. In the future, we aim to deploy our method in other centres to perform additional validation steps throughout deployment to ensure the tool’s robustness and to improve its performance. Furthermore, the current version of the tool can only be used to analyse SAX CMR scans. Future work will extend the tool to be used with long-axis cine CMR.

## Conclusions

We have developed a robust start-to-end AI-based tool for quality-controlled, automated analysis of SAX CMR scans. We implemented a state-of-the-art AI method that significantly reduces the performance drop for CMR images not seen during training. We validated our tool using over 7000 CMR cases from multiple centres and countries and showed that our method yields human-level accuracy for LV and RV segmentations for all major CMR scanner vendors, and for a wide range of cardiac disease phenotypes and acquisition protocols.

## Supplementary material

Supplementary material is available at *European Heart Journal – Digital Health*.

## Supplementary Material

ztad044_Supplementary_Data

## Data Availability

Some of the datasets presented in this study can be found in online repositories. The names of the repository/repositories and accession number(s) can be found below: The UK Biobank dataset is publicly available for approved research projects from https://www.ukbiobank.ac.uk/. The ACDC, M&Ms, and M&Ms2 datasets are publicly available to approved participants from https://acdc.creatis.insa-lyon.fr/description/databases.html, https://www.ub.edu/mnms/, and https://www.ub.edu/mnms-2/. The NHS and Duke datasets cannot be made publicly available due to restricted access under hospital ethics and because informed consent from participants did not cover public deposition of data.
