# Supplementary material for: An artificial intelligence tool for automated analysis of large-scale unstructured clinical cine cardiac magnetic resonance databases
Source: Eur Heart J Digit Health. 2023 Jul 13;4(5):370–83. doi: 10.1093/ehjdh/ztad044 (PMC10545512; doi:10.1093/ehjdh/ztad044)
Supplement: ztad044_Supplementary_Data [file ztad044_supplementary_data.docx]

**TITLE**:

An AI tool for automated analysis of large-scale unstructured clinical cine CMR databases

# Supplemental Appendix

## Supplemental Methods

1. NHS dataset acquisition, pre-processing, and quality assessment
2. Automated quality assessment of ground truth segmentations
3. Manual quality assessment of ground truth segmentations
4. Unique CMR input format
5. nnU-net and the adaptive loss function
6. Exclusion of papillary muscles
7. Post-analysis quality control
8. Cardiac biomarkers estimation

## Supplemental tables

Supplemental Table 1 – Details of CMR protocols from different scanners from the NHS database

Supplemental Table 2 – Training and validation CMR cases

Supplemental Table 3 – Original CMR image and volume formats for all datasets

Supplemental Table 4 – Automated post-analysis quality control results

Supplemental Table 5 – Inter-observer variability on a subset of the NHS data

Supplemental Table 6 – Dice scores per scanner model

Supplemental Table 7 – Comparison of our proposed model with and without QA, and against original nnUNet model trained only on the M&Ms data.

Supplemental Table 8 – Validation experiments: Dice scores per dataset with and without QC

Supplemental Table 9 – Absolute errors for cardiac biomarkers derived from the automated and manual segmentations with and without QC

## Supplemental figures

Supplemental Figure 1 – Internal (NHS) dataset curation process

Supplemental Figure 2 – Example of cases from external databases flagged by the QAgt

Supplemental Figure 3 – Original vs post-processed automated segmentations for the Duke dataset

Supplemental Figure 4 – Box plots of cardiac biomarkers per field strength group

## Supplemental Methods

### NHS dataset acquisition, pre-processing, and quality assessment

For the NHS dataset, clinical CMR scans and corresponding segmentations were automatically downloaded from the Guy's and St Thomas' NHS Foundation Trust PACS, sorted according to their acquisition sequence, and anonymised. They were then converted from a DICOM format to a NIFTI format as required by the tool, duplicate or empty ground truth segmentations were removed, end-diastolic and end-systolic frames were identified, and a thorough data quality assessment was applied to the ground truth segmentations from both frames. A summary of this process, including the number of cases discarded in each step, is provided in Supplemental Figure 1. The manual segmentations (i.e., left ventricular endocardium and myocardium and right ventricular endocardium) were acquired using the commercially available cvi42 CMR analysis software (Circle Cardiovascular Imaging Inc., Calgary, Alberta, Canada, version 5.10.1). For the NHS dataset the data quality assessment was independent to the automated quality assessment of ground truth segmentation performed on the external databases (sections B and C).

### Automated quality assessment of ground truth segmentations

We used statistical criteria together with clinical knowledge to establish the following criteria to identify potentially erroneous ground truth segmentations:

- containing unexpected labels - other than LVBP, MYO, or RVBP
- one of its ventricular volumes being equal to zero
- disagreement between image and segmentation metadata (e.g., different voxel size or orientation)
- containing outliers (i.e., voxels disconnected from the main segmentation) representing more than 10% of the total number of segmented voxels - this step was first applied to a combination of all segmented labels and then to each individual label
- any label appearing in less than 30% of segmented slices
- presence of non-segmented slices between segmented slices for any label
- negative or zero LV or RV stroke volume
- LV and RV stroke volume differences larger than 25%
- Missing basal slices on the acquisition or for segmenting

Finally, outliers representing less than 10% of the segmented voxels were automatically removed, keeping only the largest connected component for each label.

### Manual quality assessment of ground truth segmentations

The potentially erroneous ground truth segmentations flagged from section B from the Duke, ACDC, M&Ms, and M&Ms-2 datasets underwent a manual QA^gt^ that uncovered major issues in some cases. The number of excluded cases per dataset and the corresponding exclusion criteria are detailed below:

- NHS: 832 cases were excluded due to severe CMR artefacts affecting the cardiac region in any slice; ICD, pacemaker lead or sternal wires; segmentations including the atria, trabeculations, the LVOT, or the pulmonary valve; or missing or erroneous segmentations in one or more slices.
- Duke: 49 cases were excluded due to erroneous LV and/or RV segmentations. Among those cases, 40 cases were missing a basal segmentation on the LV and/or RV.
- UKBB: 85 cases were excluded due to erroneous LV and/or RV segmentations. Among those cases, 5 cases were missing a basal segmentation on the LV and/or RV.
- ACDC: 18 cases were excluded due to erroneous RV segmentations. Among those cases, 10 cases were missing a basal segmentation on the RV and 8 cases were missing the top basal slice.
- M&Ms: 21 cases were excluded due to erroneous LV segmentations. Among those cases, 15 cases were missing a basal segmentation on the LV and 2 cases were missing the top basal slice.
- M&Ms-2: 15 cases were excluded due to erroneous LV segmentations. Among those cases, 6 cases were missing a basal segmentation on the LV.

For reproducibility purposes, a list of all cases excluded from the online available databases (ACDC, M&Ms and M&Ms2) is below:

| **Database** | **Excluded IDs** |
| --- | --- |
| ACDC | patient003, patient005, patient006, patient007, patient016, patient023, patient027, patient029, patient035, patient037, patient042, patient047, patient049, patient059, patient074, patient076, patient090, patient094 |
| M&Ms | A4B5U4, A5P5W0, A6B5G9, A7F4G2, C0L7V1, C8O0P2, D1J5P6, D1S5T8, E1L7M3, E7L0N6, E9V9Z2, G9N5V9, H5N0P0, I8Z0Z6, K5M7V5 , L1Q1Z5, N7P3T8, N9P5Z0, O4O6U5, P8V0Y7, Y6Y9Z2 |
| M&Ms-2 | 008, 025, 029, 042, 045, 084, 104, 231, 242, 247, 254, 276, 294, 342, 344 |

### Unique CMR input format

Similarly to what is found in the clinic, the datasets used in this study contained different image (DICOM, MHA or NIFTI) and volume (4D or 3D) formats. Therefore, the first pre-processing step was to automatically convert all scans and segmentations to the standard 3D NIFTI format required by the tool. A summary of the diversity of original image and volume formats is shown in Supplemental Table 3.

### nnU-net and the adaptive loss function

This automated segmentation framework combines dataset- and expert-driven approaches to decide the optimal framework configuration for a given imaging dataset. The former corresponds to “rule-based” parameters which depend on dataset properties, such as imaging modality or voxel size. The latter corresponds to “fixed parameters” which have been shown to work robustly across a wide range of medical imaging segmentation applications. nnU-Net performs multiple automated pre-processing steps: it crops CMR images to a region of non-zero values, it resamples all voxels to the median voxel spacing, and it performs a z-score normalisation of the intensity values. The median spatial resolution that nnU-Net used was dx, dy, dz = (1.194, 1.194, 8) for X, Y, Z spatial resolution respectively. The same spatial resolution is used at inference time to resample the external databases.

nnU-Net uses the “U-Net” architecture as a template [1]. However, the dataset-specific U-Net configuration - kernel size, number of pooling operations, downsampling/upsampling - is determined by the image size. At this stage, computational resources (i.e., GPU RAM) are also allocated ensuring that the batch size corresponds to less than 5% of voxels in the dataset. The model is trained for 1,000 epochs (where one epoch represents an iteration over 250 mini-batches). Weights are learned via stochastic gradient descent with Nesterov momentum (µ = 0.99) and an initial learning rate of 0.01 with a ‘poly’ learning rate policy (1 − epoch/1,000)^0.9^. Deep supervision (where the contribution towards the total loss after each downsampling is halved) is used, with a combined cross-entropy and Dice loss. To tackle class imbalance, for each training image, 66.7% of patches are from random locations, while 33.3% of patches contain at least one of the foreground classes.

To tackle inconsistent image labelling (e.g., missing LVM on the ES frame) we modified nnU-Net’s loss function. The original loss function calculates the error between the predicted and manual segmentation for each label individually. This means that, when one or more labels are missing from the manual segmentation, even a perfect prediction will result in a non-zero loss. Our modification detects which labels are missing and removes these from the predicted segmentation, thus eliminating their contribution to the overall loss (e.g., if all foreground labels are missing from one patch, the loss is zero and the patch is ignored) [2, 3].

### Exclusion of papillary muscles

The LV and RV blood pool segmentations produced by the nnU-Net model are used to mask the image around those regions. Then, Otsu’s threshold method [4] is used to exclude the papillary muscles from the LV and RV pool segmentations. The threshold value used for Otsu’s method was chosen based on visual inspection of applying this method to the NHS training database. See Supplemental Figure 2 for examples of the original vs post-processed automated segmentations for the Duke dataset.

### Post-analysis quality control

Post-analysis quality control of segmentations was based on the following criteria to flag potential errors or unusual cases during automated segmentation:

- one of its ventricular volumes being equal to zero*. These errors were automatically corrected by linear interpolation of the cardiac volume.
- containing outliers (i.e., voxels disconnected from the main segmentation) representing more than 10% of the total number of segmented voxels - this step was first applied to a combination of all segmented labels and then to each individual label*. These errors were automatically corrected by applying largest connected components.
- any label appearing in less than 30% of segmented slices*. These errors were automatically corrected by deleting the corresponding label.
- presence of non-segmented slices between segmented slices for any label*
- negative or zero LV or RV stroke volume*
- LV and RV stroke volume differences larger than 25%*
- LV/RV mass differences between LV and RV larger than 15%
- For full short axis sequences, maximum change of volume between adjacent phases greater than 25 mL

Supplemental Table 4 shows the percentage of automated segmentations that were flagged up by this post-analysis quality control (QC) for each dataset. Overall, cases were mostly flagged up due to large (>25%) stroke volume differences between the LV and the RV.

Note that the criteria marked with asterisks are common between QA^gt^ and QC. We refer the reader to Supplemental Method B for more details of the criteria for automated quality assessment of ground truth segmentations.

### Cardiac biomarkers estimation

Cardiac biomarkers were directly computed from segmentation masks for the internal and external database. We use this approach as some biomarkers were not directly available on all external databases. For the NHS training database, we perform a statistical analysis to compare the cardiac biomarkers directly outputted from CVI42 to biomarkers derived from the manual annotated masks and we found no statistically significant difference and a bias less than 5 mL for EDV and ESV and less than 1% for EF.

## Supplemental tables

**Supplemental Table 1 – Details of CMR protocols from different scanners from the NHS database**

|  | **Siemens Aera 1.5 T** | **Siemens Biograph mMR 1.5T** | **Philips Achieva 1.5T/3.0T** | **Philips Ingenia 1.5T** |
| --- | --- | --- | --- | --- |
| **Scanning sequence** | GR | GR | GR | GR |
| **Sequence variant** | OSP, SK | OSP, SK | SK | SK |
| **Echo time (TE), mm** | 1.03 – 3.17 | 1.44 – 1.50 | 1.28 – 1.67 | 1.27 – 1.55 |
| **Repetition time (TR), mm** | 10.08 – 85.87 | 36.85 – 129.20 | 2.56 – 3.34 | 2.54 – 3.09 |
| **Flip angle, °** | 15 - 57 | 25 | 60 | 10 – 30 |
| **Slice thickness (mm)** | 6 - 10 | 8 | 8 - 10 | 8 - 10 |
| **Pixel spacing, mm^2^** | 1.52 – 2.39 | 1.41 – 1.56 | 0.87 – 1.46 | 0.86 – 1.34 |
| **Number of frames** | 11 - 50 | 43 - 50 | 15 - 30 | 40 – 50 |

Table shows details of CMR protocols for the different scanner used on the NHS database. Different scanning sequence and sequence variant values are reported in full. Other parameters are as minimum/maximum value.

**Supplemental Table 2 – Training and validation CMR cases**

| **Dataset** | **Train** | **Validation, before QA^gt^** | **Validation, after QA^gt^** |
| --- | --- | --- | --- |
| NHS | 2793* | 414* | 414* |
| Duke | 0 | 1319 | 1270 |
| UKBB | 0 | 4872 | 4787 |
| ACDC | 0 | 150 | 132 |
| M&Ms | 0 | 375 | 354 |
| M&Ms-2 | 0 | 360 | 345 |

CMR cases containing segmentations that were used for training and validation (before and after QA^gt^). QA^gt^: data quality assessment of ground truth segmentations.

* see details on the ground-truth data assessment of NHS data in the flow chart in Supplemental Figure 1.

**Supplemental Table 3 – Original CMR image and volume formats for all datasets**

| **Dataset** | **Original image format** | | | **Original volume format** | |
| --- | --- | --- | --- | --- | --- |
|  | **DICOM** | **NIFTI** | **MHA** | **4D** | **3D** |
| NHS | x |  |  | x |  |
| Duke | x |  |  | x |  |
| UKBB | x |  |  | x |  |
| ACDC |  |  | x |  | x |
| M&Ms |  | x |  | x |  |
| M&Ms-2 |  | x |  |  | x |

**Supplemental Table 4 – Automated post-analysis quality control results**

|  | **Dataset** | | | | | |
| --- | --- | --- | --- | --- | --- | --- |
| **Post-analysis QC** | **NHS** | **Duke** | **UKBB** | **ACDC** | **M&Ms** | **M&Ms-2** |
| Global outlier >10% [%]* | 0.00 | 0.00 | 0.08 | 0.00 | 0.00 | 0.29 |
| LVBP outlier >10% [%]* | 0.00 | 0.00 | 0.04 | 1.22 | 0.00 | 0.58 |
| LVM outlier >10% [%]* | 0.24 | 0.31 | 0.02 | 0.00 | 0.31 | 0.00 |
| RVBP outlier >10% [%]* | 0.00 | 0.16 | 0.04 | 1.22 | 0.00 | 0.29 |
| SV differences > 25% [%] | 6.35 | 9.51 | 7.08 | 11.22 | 10.99 | 14.78 |
| Flagged up cases [%] | 6.59 | 9.83 | 7.21 | 12.44 | 11.30 | 15.36 |

The first five rows show the percentage of cases that were flagged up for clinician review due to each of the criteria. Since some cases were flagged up after fulfilling multiple criteria, the last row indicates the percentage of cases that were flagged up at least once.

* see Supplemental Method G for a definition of outlier pixels in segmentations.

**Supplemental Table 5 – Inter-observer variability on a subset of the NHS data**

| **Absolute errors** |  |  |  |  |
| --- | --- | --- | --- | --- |
|  | **O1 vs O2**  **(n = 50)** | **O1 vs O3**  **(n = 50)** | **O2 vs O3**  **(n = 50)** | **Auto vs Manual**  **(n = 50)** |
| LVEDV [mL] | 6.98 (7.50) | 6.52 (7.47) | 6.42 (8.47) | 6.98 (7.31) |
| LVESV [mL] | 5.75 (8.29) | 5.68 (9.68) | 7.06 (9.06) | 6.25 (9.01) |
| LVM [g] | 8.78 (8.64) | 8.16 (9.14) | 8.20 (8.96) | 8.88 (8.99) |
| RVEDV [mL] | 8.18 (7.63) | 7.81 (8.25) | 8.31 (9.06) | 8.36 (7.99) |
| RVESV [mL] | 6.10 (8.97) | 5.99 (7.92) | 6.15 (7.79) | 6.5 (8.01) |

Top table: median (interquartile range) absolute errors for cardiac biomarkers for the NHS inter-observer dataset and automated for the same subset of cases used for NHS inter-observer variability. LVBP: left ventricular blood pool, MYO: left ventricular myocardium, RVBP: right ventricular blood pool, LVBP: LV blood pool, MYO: LV myocardium, RVBP: RV blood pool, ED: end diastole, ES: end systole.

**Supplemental Table 6 – Dice scores per scanner model**

|  | **LVBP** | | **MYO** | | **RVBP** | |
| --- | --- | --- | --- | --- | --- | --- |
| **Scanner model** | **ED** | **ES** | **ED** | **ES** | **ED** | **ES** |
| Philips Achieva (1.5T, 3.0T)  (n=297) | 0.95 (0.02) | 0.91 (0.05) | 0.85 (0.05) | 0.85 (0.05) | 0.93 (0.03) | 0.88 (0.05) |
| Siemens Aera (1.5T)  (n=4936) | 0.94 (0.02) | 0.88 (0.05) | 0.82 (0.05) | 0.84 (0.04) | 0.90 (0.03) | 0.83 (0.06) |
| Siemens Avanto (1.5T)  (n=272) | 0.93 (0.04) | 0.87 (0.07) | 0.82 (0.04) | 0.83 (0.05) | 0.91 (0.04) | 0.85 (0.08) |
| Siemens Biograph mMR (3.0T)  (n=7) | 0.95 (0.01) | 0.91 (0.03) | 0.86 (0.03) | N/A* | 0.92 (0.02) | 0.87 (0.04) |
| General Electric Excite (1.5T)  (n=58) | 0.95 (0.02) | 0.89 (0.07) | 0.85 (0.05) | 0.87 (0.04) | 0.92 (0.04) | 0.88 (0.06) |
| General Electric HDxt (3.0T)  (n=14) | 0.95 (0.02) | 0.92 (0.04) | 0.83 (0.03) | 0.86 (0.03) | 0.89 (0.04) | 0.86 (0.05) |
| Philips Ingenia (1.5T)  (n=114) | 0.95 (0.02) | 0.92 (0.04) | 0.86 (0.04) | 0.84 (0.05) | 0.92 (0.03) | 0.88 (0.05) |
| Canon Orian (1.5T)  (n=25) | 0.94 (0.03) | 0.91 (0.04) | 0.83 (0.03) | 0.86 (0.03) | 0.92 (0.03) | 0.91 (0.03) |
| Siemens Sola (1.5T)  (n=146) | 0.93 (0.03) | 0.86 (0.08) | 0.82 (0.04) | 0.86 (0.04) | 0.91 (0.03) | 0.86 (0.05) |
| Siemens Symphony (1.5T)  (n=101) | 0.95 (0.02) | 0.92 (0.05) | 0.86 (0.05) | 0.86 (0.05) | 0.91 (0.06) | 0.87 (0.06) |
| Siemens Trio (3.0T)  (n=3) | 0.97 (0.00) | 0.92 (0.04) | 0.87 (0.01) | 0.86 (0.02) | 0.89 (0.03) | 0.82 (0.08) |
| Siemens Verio (3.0T)  (n=47) | 0.94 (0.02) | 0.84 (0.09) | 0.82 (0.03) | N/A* | 0.91 (0.04) | 0.86 (0.05) |
| Siemens Vida (3.0T)  (n=581) | 0.93 (0.02) | 0.86 (0.07) | 0.83 (0.03) | 0.85 (0.03) | 0.91 (0.04) | 0.86 (0.05) |

Median (interquartile range) values for each scanner model and label (LVBP, MYO, and RVBP) in ED and ES. *No manual segmentations contained the myocardium in ES. LVBP: LV blood pool, MYO: LV myocardium, RVBP: RV blood pool, ED: end diastole, ES: end systole.

**Supplemental Table 7 – Comparison of our proposed model with and without QA, and against original nnUNet model trained only on the M&Ms data.**

| **Model** | **Dataset** | **LVBP [%]** | **MYO [%]** | **RVBP [%]** | **Average [%]** |
| --- | --- | --- | --- | --- | --- |
| Ours with QA^gt^ | NHS | 94.3 (4.0) | 85.5 (4.4) | 90.8 (5.4) | 91.3 (7.5) |
| Ours without QA^gt^ | NHS | 93.1 (4.76)* | 83.6 (5.25)* | 89.2 (4.43)* | 88.6 (7.9)* |
| M&Ms | NHS | 92.2 (5.1)* | 81.7 (4.7)* | 88.1 (5.7)* | 87.3 (8.2)* |

Dice scores are shown as median (interquartile range) percentages for each the NHS validation dataset, including values per label and their average. First row shows the results for our proposed method with QA^gt^, second row shows the results for our proposed method without QA^gt^ and third row shows the results for the comparative state-of-the-art method trained only on the M&M database. Comparisons between the Dice scores of the NHS validation cases for our proposed method with QA^gt^ and the two comparative approaches (without QA^gt^ and original M&M model) were performed using Mann-Whitney U tests. Asterisks indicate statistically significant differences for each label, where * = p < 0.01. LVBP: LV blood pool, MYO: LV myocardium, RVBP: RV blood pool.

**Supplemental Table 8 – Validation experiments: Dice scores per dataset with and without QC.**

|  | **Dice scores without QC** | | | | **Dice scores with QC** | | | |
| --- | --- | --- | --- | --- | --- | --- | --- | --- |
| **Dataset** | **LVBP** | **MYO** | **RVBP** | **Average** | **LVBP** | **MYO** | **RVBP** | **Average** |
| NHS | 93.49 (3.76) | 84.66 (4.25) | 89.80 (4.43) | 90.22 (5.27) | 94.3 (3.01) | 85.5 (4.22) | 90.8 (4.41) | 91.3 (5.24) |
| Duke | 89.22 (6.80)* | 82.20 (4.09)* | 87.90 (5.92)* | 87.72 (6.52)* | 91.3 (6.50) | 83.0 (3.81) | 89.3 (5.83) | 89.2 (6.33) |
| UKBB | 90.79 (4.81) | 82.53 (4.86) | 86.64 (5.89) | 86.65 (6.21) | 91.8 (4.92) | 83.0 (4.53) | 87.8 (5.82) | 87.4 (5.95) |
| ACDC | 93.40 (5.38)* | 86.39 (4.69)* | 89.60 (6.63)* | 89.80 (6.31) | 95.5 (4.91) | 87.4 (3.52) | 91.8 (6.04) | 90.6 (5.95) |
| M&Ms | 91.62 (5.72)* | 84.51 (4.82)* | 88.38 (6.90) | 88.17 (6.56) | 93.4 (5.41) | 85.4 (4.71) | 90.4 (5.83) | 89.4 (6.34) |
| M&Ms-2 | 93.51 (4.02)* | 85.03 (5.17) | 89.76 (5.47) | 89.44 (6.02) | 94.6 (4.01) | 86.0 (5.02) | 90.9 (5.73) | 90.3 (6.31) |

Left table: median (interquartile range) values for each validation dataset, including values per label and their average, excluding cases that did not pass the QA^gt^. Right table: median (interquartile range) values for each validation dataset, including values per label and their average, including cases that passed the QA^gt^. LVBP: left ventricular blood pool, MYO: left ventricular myocardium, RVBP: right ventricular blood pool, QA^gt^: data quality assessment of ground truth segmentations. Comparisons between the Dice scores with and without QA^gt^ were performed using Mann-Whitney U tests. Bonferroni correction for multiple comparisons. Asterisks indicate statistically significant differences for each label after correction (20 tests), where * = p < 0.01/20.

**Supplemental Table 9 – Absolute errors for cardiac biomarkers derived from the automated and manual segmentations with and without QC**

|  | **Absolute errors for cardiac biomarkers without QC** | | | | | | | **Absolute errors for cardiac biomarkers with QC** | | | | | | |
| --- | --- | --- | --- | --- | --- | --- | --- | --- | --- | --- | --- | --- | --- | --- |
|  | **LV** | | | | **RV** | | | **LV** | | | | **RV** | | |
| **Dataset** | **EDV [mL]** | **ESV [mL]** | **EF**  **[%]** | **LVM**  **[g]** | **EDV [mL]** | **ESV [mL]** | **EF**  **[%]** | **EDV [mL]** | **ESV [mL]** | **EF**  **[%]** | **LVM**  **[g]** | **EDV [mL]** | **ESV [mL]** | **EF**  **[%]** |
| NHS | 8.96 (8.04) | 7.97 (6.93) | 3.38 (2.83) | 11.83 (9.93) | 11.06 (9.43) | 8.49 (7.41) | 4.17 (3.39) | 6.7 (13.5) | 6.3 (12.9) | 3.4 (4.3) | 8.9 (17.9) | 8.5 (15.8) | 6.4 (12.5) | 4.2 (5.4) |
| Duke | 7.25 (6.62) | 6.62 (6.24) | 5.32 (4.73) | 16.75 (13.70)* | 8.49 (8.58) | 6.33 (6.17)* | 5.37 (4.55) | 5.7 (11.0) | 5.0 (9.7) | 5.3 (7.1) | 13.3 (17.2) | 6.0 (11.8) | 4.5 (9.1) | 5.2 (6.8) |
| UKBB | 9.36 (7.98)* | 9.39 (6.62)* | 4.67 (3.39) | 7.82 (6.27)* | 11.51 (9.37)* | 10.64 (8.42)* | 5.31 (3.92) | 7.3 (12.5) | 8.4 (9.6) | 4.7 (4.2) | 6.6 (12.8) | 9.2 (14.7) | 8.9 (12.5) | 5.3 (5.9) |
| ACDC | 4.51 (3.39) | 7.51 (6.32)* | 4.56 (4.31) | 9.04 (8.25)* | 8.17 (7.54) | 9.94 (10.44)* | 6.00 (5.28) | 3.6 (5.8) | 5.3 (8.0) | 4.6 (5.1) | 6.8 (12.7) | 6.1 (11.2) | 5.7 (11.1) | 5.6 (7.6) |
| M&Ms | 9.18 (8.78)* | 7.00 (6.29)* | 4.49 (3.93) | 10.73 (10.42)* | 11.21 (10.24) | 8.82 (10.83)* | 5.66 (5.22) | 6.2 (13.4) | 5.1 (9.8) | 4.2 (5.4) | 8.2 (16.6) | 8.4 (16.9) | 5.9 (13.7) | 5.6 (7.3) |
| M&Ms-2 | 8.29 (7.46)* | 7.22 (6.29) | 3.47 (3.18) | 9.39 (8.53)* | 13.00 (13.98) | 11.68 (12.20)* | 5.98 (4.98) | 6.3 (9.6) | 5.5 (8.4) | 3.4 (4.2) | 7.3 (14.2) | 8.7 (16.5) | 8.3 (13.9) | 5.9 (7.0) |
| Average | 7.93 | 7.62 | 4.32 | 10.93 | 10.57 | 9.32 | 5.42 | 5.97 | 5.93 | 4.27 | 8.52 | 7.82 | 6.62 | 5.30 |

Left: Median (interquartile range) absolute errors for cardiac biomarkers derived from the automated and manual segmentations for each validation dataset, excluding cases that did not pass the QA^gt^. Right: Median (interquartile range) absolute errors for cardiac biomarkers derived from the automated and manual segmentations for each validation dataset, including cases that passed the QA^gt^. QA^gt^: data quality assessment of ground truth segmentations. Last row shows a summary of mean error values across all datasets. Comparisons between absolute errors for cardiac biomarkers with and without QA^gt^ were performed using Mann-Whitney U tests. Bonferroni correction for multiple comparisons. Asterisks indicate statistically significant differences for each label after correction (35 tests), where * = p < 0.01/35.

## Supplemental figures


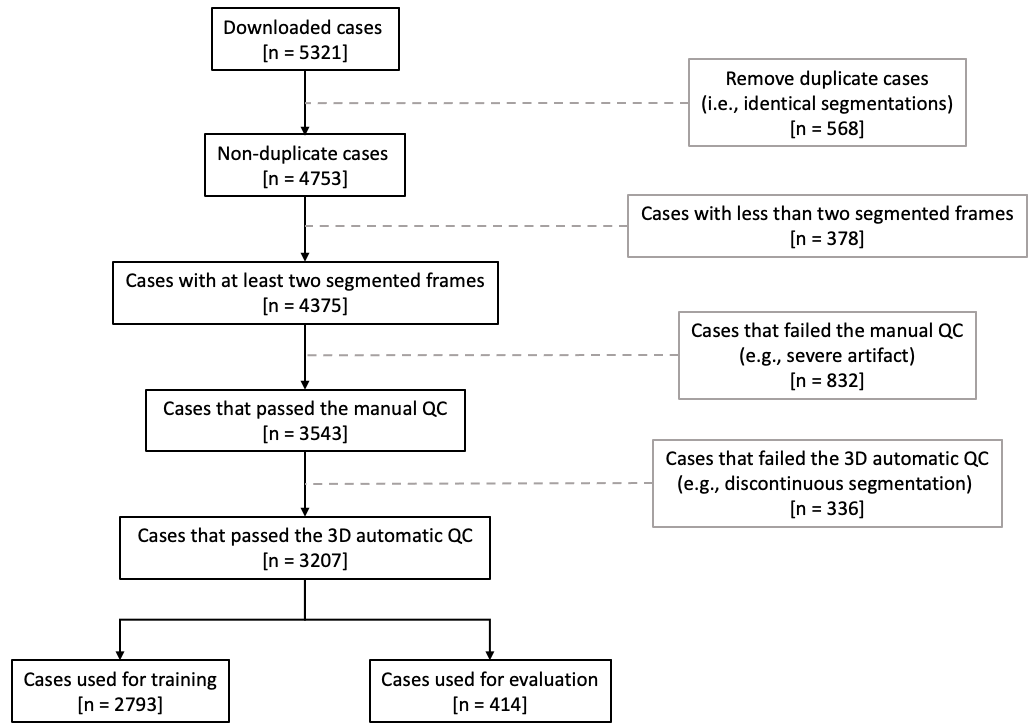


**Supplemental Figure 1 – Internal (NHS) dataset curation process**: Number of NHS cases discarded during download, pre-processing, and quality assessment (QA^gt^).


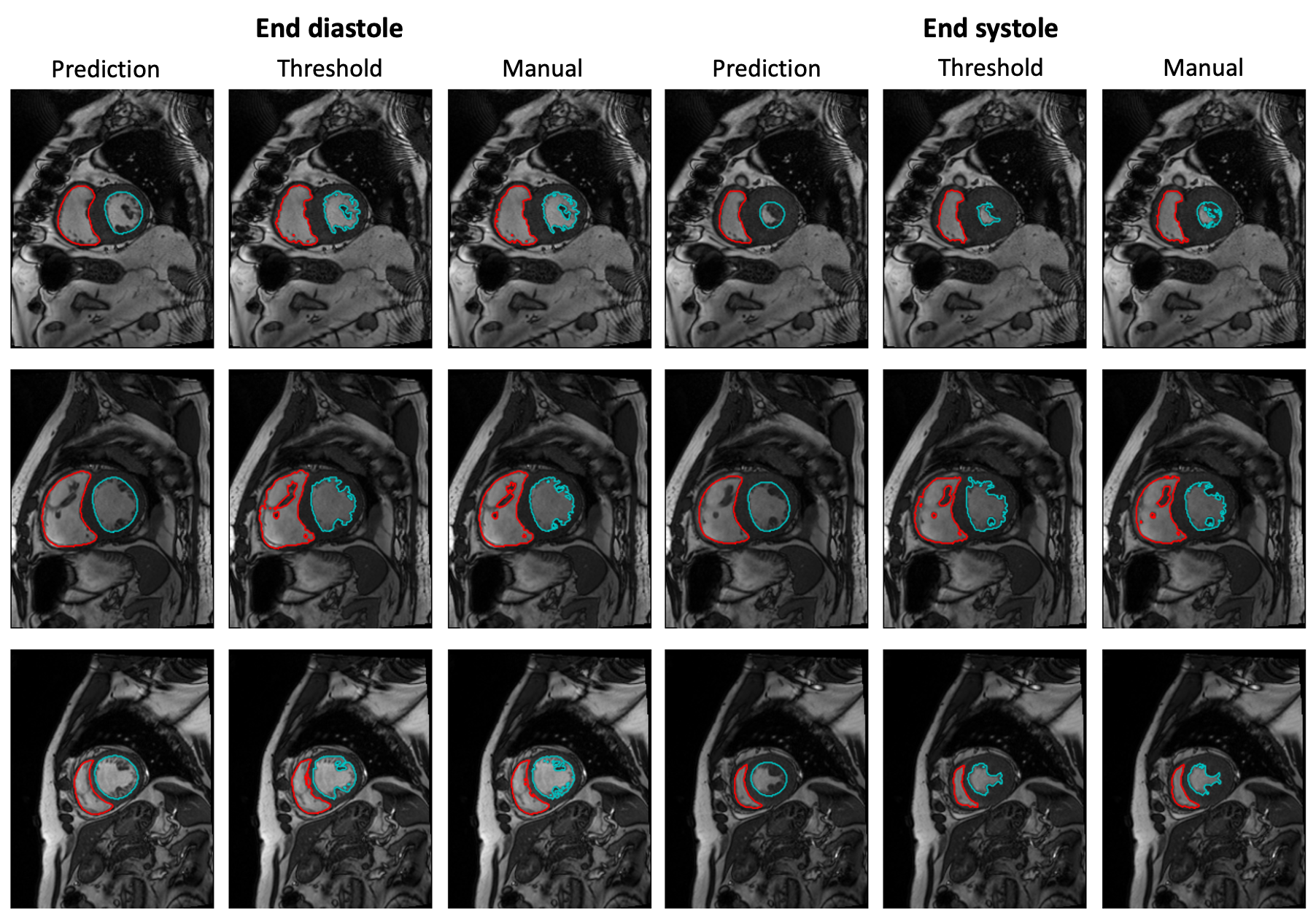
**Supplemental Figure 2 – Original vs post-processed automated segmentations for the Duke dataset**: Comparison between original and post-processed automated segmentations for the Duke dataset using Otsu’s threshold method. Each row depicts end-diastolic (left) and end-systolic (right) segmentations located in the middle of the heart for a random case.


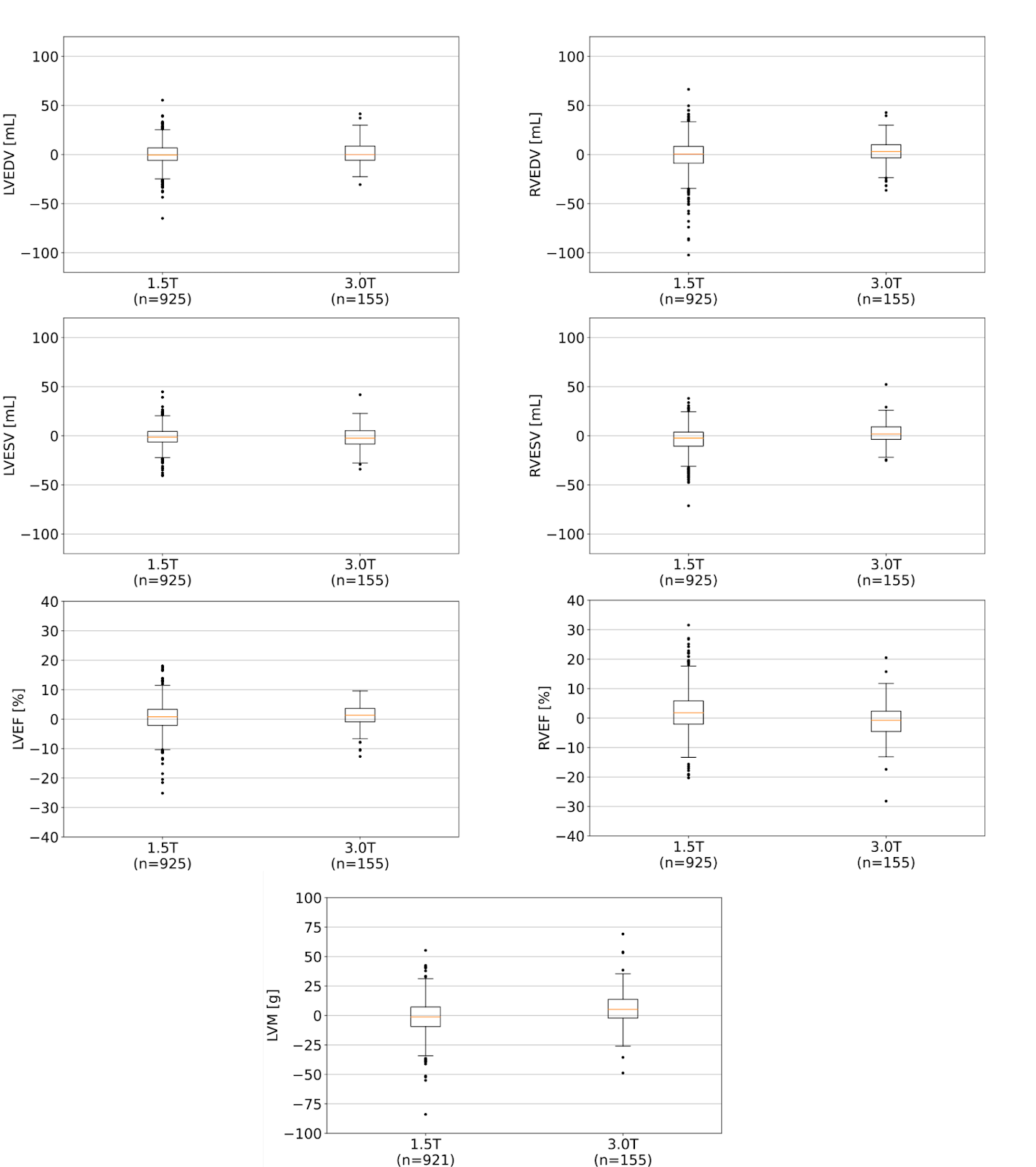


**Supplemental Figure 3 – Box plots of cardiac biomarkers per field strength group**: Box plots of manually and automatically derived cardiac biomarkers for each magnetic field strength group. Abbreviations as in Figure 2.

**Supplemental Figure 4 – Example of ground-truth cases from external databases flagged by our quality assessment of ground-truth data (QA^gt^).** Selection of excluded cases for the online external databases (M&Ms, M&Ms-2 and ACDC). The majority of cases were excluded because of missing basal slices, with the short-axis stack not continuing into the atria (examples show the top slices of case A5P5W0 from M&Ms and case patient090 from ACDC). Other issues raised were missing segmentation (see the clear myocardial rim for both LV and RV in the ES in slice 2 and ED in slice 3 of case 008 from M&Ms-2, missing RV segmentation in the ES frame from Patient090, LV segmentation in the ES frame of case A5P5W0, the missing RVOT segmentation in the ES frame of case Y6Y972 and the LV segmentation in the ED frame of case 025 from M&Ms-2), lastly we automatically raised SV differences of larger than 25% between RV and LV (here case A4B5UA, from M&Ms)References

1. Ronneberger O, Fischer P, Brox T. U-net: Convolutional networks for biomedical image segmentation. In: Lecture Notes in Computer Science (including subseries Lecture Notes in Artificial Intelligence and Lecture Notes in Bioinformatics). 2015. p. 234–41.

2. Tilborghs S, Bertels J, Robben D, Vandermeulen D, Maes F. The Dice Loss in the Context of Missing or Empty Labels: Introducing Phi and epsilon. 2022. p. 527–37.

3. Petit O, Thome N, Charnoz A, Hostettler A, Soler L. Handling Missing Annotations for Semantic Segmentation with Deep ConvNets. Deep Learning in Medical Image Analysis and Multimodal Learning for Clinical Decision Support: 4th International Workshop, DLMIA 2018, and 8th International Workshop, ML-CDS 2018, Held in Conjunction with MICCAI 2018, Granada, Spain, September 20, 2018, P; 2018. p. 20–8.

4. Otsu N. A Threshold Selection Method from Gray-Level Histograms. IEEE Trans Syst Man Cybern. 1979;C:62–6.
